# Comparison of microbiota and allergen profile in house dust from homes of allergic and non-allergic subjects- results from the GUSTO study

**DOI:** 10.1186/s40413-018-0212-5

**Published:** 2018-12-05

**Authors:** Evelyn Xiu Ling Loo, Lamony Jian Ming Chew, Atiqa Binte Zulkifli, Le Duc Huy Ta, I-Chun Kuo, Anne Goh, Oon Hoe Teoh, Hugo Van Bever, Peter D. Gluckman, Fabian Yap, Kok Hian Tan, Yap Seng Chong, Bee Wah Lee, Lynette Pei-chi Shek

**Affiliations:** 1 Singapore Institute for Clinical Sciences (SICS), Agency for Science, Technology and Research (A*STAR), Singapore, Singapore; 2 Department of Paediatrics, Yong Loo Lin School of Medicine, National University of Singapore, Singapore, Singapore; 3 Allergy Service, Department of Paediatrics, KK Women’s and Children’s Hospital, Singapore, Singapore; 4 Department of Obstetrics & Gynaecology, Yong Loo Lin School of Medicine, National University of Singapore, Singapore, Singapore; 5 Growth, Development and Metabolism Programme, Singapore Institute for Clinical Sciences (SICS), Agency for Science, Technology and Research (A*STAR), Singapore, Singapore; 6 Liggins Institute, University of Auckland, Auckland, New Zealand; 7 Khoo Teck Puat-National University Children’s Medical Institute, National University Hospital, National University Health System, Singapore, Singapore; 8 Department of Maternal Fetal Medicine, KK Women’s and Children’s Hospital, Singapore, Singapore; 9 Department of Endocrinology, KK Women’s and Children’s Hospital, Singapore, Singapore; 10 Respiratory Medicine Service, Department of Paediatrics, KK Women’s and Children’s Hospital, Singapore, Singapore

## Abstract

**Background:**

The prevalence of allergic diseases, such as asthma, allergic rhinitis, eczema and food allergy, has been increasing worldwide, as shown in a large number of studies, including the International Study of Asthma and Allergies in Childhood (ISAAC). However, there is significant variation in the prevalence of these diseases in different regions, suggesting that there may be location-specific factors such as environment and microbial exposure affecting allergic disease prevalence. Hence, in this study we determine if there is a difference in microbiota composition and allergen concentration of household dust collected from the homes of non-allergic and allergic subjects from the Growing Up in Singapore Towards Healthy Outcomes (GUSTO) cohort.

**Methods:**

From the Growing Up in Singapore Towards Healthy Outcomes (GUSTO) cohort, 25 allergic subjects and 25 non-allergic subjects were selected at the year 5.5 follow up. Definitions of allergic outcomes were standardized in the questionnaires administered at 3, 6, 9, 12, 15, 18, 24, 36, 48 and 60 months to ensure consistency during interviews and home visits. Allergen sensitization was determined by skin prick testing (SPT) at 18, 36 and 60 months. Dust samples were collected from the subject’s bed, sofa, and play area. DNA extraction was carried out and V3-V4 hypervariable regions of bacterial 16S rRNA gene were sequenced. Protein extraction was performed and allergens assayed by using multiplex assay and ELISA.

**Results:**

The most abundant phyla in house dust were Actinobacteria (29.8%), Firmicutes (27.7%), and Proteobacteria (22.4%). Although there were no differences in bacteria abundance and diversity between house dust samples of allergic and non-allergic subjects, the relative abundance of *Anaplasmataceae*, *Bacteroidaceae*, and *Leptospiraceae* were significantly higher in dust samples of allergic subjects as compared to non-allergic subjects in 2 or more locations. The concentration of *Der p* 1 was significantly lower in bed dust samples of allergic subjects (Median [Interquartile range], 174 ng/g [115–299 ng/g]) as compared to non-allergic subjects (309 ng/g [201–400 ng/g]; *P* < 0.05). The concentration of tropomyosin was significantly higher in sofa dust samples of allergic subjects (175 ng/g [145–284 ng/g] as compared to non-allergic subjects (116 ng/g [52.8–170 ng/g]; *P* < 0.05).

**Conclusion:**

In conclusion, we found a differential microbiota and allergen profile between homes of allergic and non-allergic subjects.

**Trial registration:**

NCT01174875 Registered 1 July 2010, retrospectively registered.

**Electronic supplementary material:**

The online version of this article (10.1186/s40413-018-0212-5) contains supplementary material, which is available to authorized users.

## Background

The prevalence of allergic diseases such as asthma, allergic rhinitis, eczema and food allergy has been increasing worldwide, as shown in a large number of studies, including the International Study of Asthma and Allergies in Childhood (ISAAC) [1]. However, there is significant variation in the prevalence of these diseases in different regions, suggesting that there may be location-specific factors such as environment and microbial exposure affecting allergic disease prevalence [2]. The difference in rates of allergic diseases between genetically similar populations in different parts of the world further emphasizes the differences in environmental risk factors that may play an influential role in the development of allergy [3]. However, most of the literature in the field focused on gut microbiota, with limited information on environmental microbiota and its influence on disease development [4–9].

The hygiene hypothesis states that cleaner environments result in a decrease in “natural” Th1/ Treg-trophic microbial signals resulting in a shift to a pro-inflammatory Th2 environment [10]. It is thought that a lack of exposure to microbial or infectious agents increases the susceptibility to atopic diseases such as atopic dermatitis, allergic rhinitis and/ or allergic asthma by suppressing the natural development of the immune system [11]. However, recent literature in the field has been conflicting. While there are various studies in different populations demonstrating an inverse relationship between microbial exposure and development of allergic symptoms such as atopy and asthma [12–14], others showed a positive correlation between microbial exposure and allergy [15–18]. A study conducted in Germany that analyzed the mattress dust from 489 children showed that increased exposure to a rich microbial environment protects against atopy and asthma in children aged between 6 and 13 years old living in rural and sub-urban environments [12] while another study conducted in USA that recruited children from 308 homes showed an increase in bacterial diversity/abundance in the homes of asthmatic patients, aged 2.4 ± 0.9 years (mean ± SD) [19].

To our knowledge, most of these studies that specifically investigated the bacterial composition of house dust have dust sampling carried out at a single site, mainly from the bed [12, 20, 21]. In addition, the studies are mostly conducted in the Western countries [12, 14, 19, 22–25] where lifestyle and environmental factors differ from Asian countries. Hence in this study, we determined if there is a difference in microbiota composition, and allergen concentration of household dust collected from 3 different sites in the houses of 25 non-allergic and 25 allergic subjects from the Growing Up in Singapore towards Healthy Outcomes (GUSTO) birth cohort at the 5.5 year follow up.

## Methods

### Study design and subjects

From the Growing Up in Singapore Towards Healthy Outcomes (GUSTO) cohort, we conducted a pilot study with random sampling of houses of 25 allergic subjects and 25 non-allergic subjects at the year 5.5 follow up. The methodology of the GUSTO study has been described previously [26]. Briefly, we recruited healthy pregnant mothers who agreed to enroll their offspring for future follow-up. Interviewers gathered information on demographic characteristics, family history of allergy, social data and lifestyle factors. Definitions of allergic outcomes were standardized in the questionnaires administered at 3, 6, 9, 12, 15, 18, 24, 36, 48 and 60 months to ensure consistency during interviews and home visits. We used the ISAAC modified questionnaire as used in other studies [27]. Physician-diagnosed atopic eczema was based on a positive answer to the written question: “Has your child ever been diagnosed with eczema?”, “Wheezing” was based on positive answers to “Has your child ever wheezed?” and “Has your child ever been prescribed with nebulizer/inhaler?”, while “rhinitis” was based on a positive response to the question “Has your child ever had sneezing, running nose, blocked or congested nose, snoring or noisy breathing during sleep or when awake that has lasted for 2 or more weeks duration?”. Allergen sensitization was determined by skin prick testing (SPT) to house dust mite allergens (*Dermatophagoides pteronyssinus*, *Dermatophagoides farinae,* and *Blomia tropicalis*) and food allergens (egg, peanut and cow’s milk) at 18, 36 and 60 months. At 60 months, skin prick testing was also carried out to shrimp and crab allergens. The allergens for skin prick testing were obtained from Greer Laboratories (Lenoir, NC, USA), except for *B. tropicalis*, which was obtained from our laboratory. Tests were interpreted as positive if the wheal was at least 3 mm, and a child was considered as SPT-positive if any one or more of the individual tests was positive with a positive reaction to the positive control (histamine) and a negative reaction to the negative control (saline). Allergic subjects have a positive SPT to at least one of the tested allergens at year 5 and have ever given positive replies to questions on allergic outcomes. Non-allergic subjects have no positive SPT and answered “no” to all questions on allergy in the first 5 years of life. Ethics approval was obtained from the Domain Specific Review Board of Singapore National Healthcare Group and the Centralised Institutional Review Board of SingHealth. Conduct of this study was based on the guidelines in the Declaration of Helsinki. Informed consent was obtained from all participants.

### Sample collection and processing

Dust samples were collected from a random sampling of houses of 25 allergic subjects, and 25 non-allergic subjects at year 5.5. Singapore is an urban city and the samples were collected from the subject’s bed, sofa, and 2 m by 1 m play area, using vacuum cleaners (Dyson DC63; Dyson, UK) with 40 μm nylon mesh DUSTREAM® filters (Indoor Biotechnologies, India). Each area was vacuumed for 4 min. Debris were manually removed from the dust samples and all samples were then stored at − 80 °C until further processing.

For DNA analyses, the samples were processed using PowerFood® Microbial DNA Isolation Kit (Mobio Laboratories, Inc., USA), with slight modification in cell lysis procedure as compared to the manufacturer’s protocol. Briefly, the samples were resuspended in 450 μl Solution PF1 and disrupted in MicroBead Tube using FastPrep-24 instrument (MP Biomedicals, USA) for 20 s at 4 m/s twice. Total DNA was extracted and stored at − 30 °C until sequencing of bacteria 16S rRNA.

For proteomic analyses, the samples were extracted in Tris-buffered-saline, containing 0.05% Tween-20 (Sigma-Aldrich, USA), and Halt Protease and Phosphatase Inhibitor Cocktail (Thermo Fisher Scientific, USA) at a ratio of 1 mg to 50 μl. The samples were rotated at 4 °C for 2 h, and the supernatants were stored at − 80 °C until measurement of various indoor allergens.

### Bacterial 16S rRNA gene amplification and sequencing

Bacterial V3-V4 hypervariable regions of the 16S rRNA gene were sequenced according to the Illumina 16S metagenomics sequencing library preparation protocol with some modifications. Briefly, bacterial V3-V4 hypervariable regions of the 16S rRNA gene were amplified using the bacteria primer 340F (5’-CCTACGGGNGGCWGCAG-3′) and reverse primer 805R (5′- GACTACNVGGGTATCTAATCC -3′), flanked with Illumina overhang adapters [28, 29]. PCR amplifications were performed in a final volume of 25 μl, containing 1.25 μl of 10 μM each primer, 1 μl of template DNA and 12.5 μl of Q5® Hot Start High-Fidelity 2× Master Mix (New England Biolabs, USA). The PCR conditions were as follows: 98 °C for 30 s; 30 cycles of 98 °C for 10 s, 71.6 °C for 30 s, 72 °C for 30s; followed by 72 °C for 10 mins. A subsequent limited-cycle amplification step was performed to attach multiplexing indices and Illumina adapter sequences to the amplicons. Amplicons from each sample was subsequently quantified by qPCR using the KAPA library quantification kit (KAPA Biosystems, USA) and pooled in equimolar concentrations into a single library. The pooled libraries were loaded into MiSeq instrument (Illumina, USA) for cluster generation and sequencing according to specifications to generate paired-end reads of 250 bp each. The sequencing data were matched with Greengenes database using Illumina BaseSpace 16S Metagenomics Application.

### Measurement of indoor allergens

Concentration of *Blo t* 5 was measured using sandwich enzyme-linked immunosorbent assay (ELISA) as previously described [30], with slight modification. Briefly, microtiter plate was coated overnight at 4 °C with 2 μg/ml of mouse anti-*Blo t* 5 monoclonal antibody in 0.1 M sodium bicarbonate buffer, pH 8.3. After blocking, 50 μl of dust sample was added to the wells and incubated at 4 °C overnight. Guinea pig anti-*Blo t* 5 serum (1:2000), and horseradish peroxidase-conjugated goat anti-guinea pig IgG (1:10,000; Sigma-Aldrich, USA) were used as secondary antibodies and detection antibodies respectively. Signal was developed by addition of TMB substrate (eBioscience, Inc., USA).

Concentrations of *Der p* 1, *Der f* 1, mite group 2, *Fel d* 1 and *Can f* 1 were measured using Multiplex Array for Indoor Allergens (MARIA™; Indoor Biotechnologies, UK), as per manufacturer’s instructions. Concentration of tropomyosin was measured using Tropomoysin ELISA kit (Indoor Biotechnologies, UK), as per manufacturer’s instructions. Results were reported as nanogram of allergen per gram of dust sample. The lower limits of detection were for 3.05 ng/g for *Der p* 1, *Der f* 1, and *Can f* 1; 1.22 ng/g for mite group 2 and *Fel d* 1; 1.95 ng/g for *Blo t* 5; 5 ng/g for Tropomoysin.

### Statistical analysis

For DNA analysis, data were analyzed using CRAN software with ggplot2 package for analysis of phyla composition, and vegan package for comparison of relative abundance of bacterial families. Difference in relative abundance of bacterial families with IndVal value > 0.6 and *P*-value < 0.05 were considered significant. Data were also analyzed using QIIME version 1.9.1 for alpha diversity, and plotted using GraphPad Prism 5 software (GraphPad Software, Inc., USA). To compare the bacterial diversity of house dust samples of allergic subjects and non-allergic subjects, Shannon and Simpson’s alpha diversity indices of the samples were compared using Mann-Whitney U test. Differences with *P*-value < 0.05 were considered significant.

For proteomic analysis, data were plotted and analyzed using GraphPad Prism 5 software. The data were compared using Mann–Whitney U test. Differences with *P*-value < 0.05 were considered significant.

## Results

### Study population

There were differences in gender and maternal education levels between the groups. There were significantly more boys and mothers with a higher education level in the allergic group (Table 1).

**Table 1:** Comparison of demographic variables and cleaning frequency between the groups

Variable	Allergic group	Non-allergic group	*p*-value
	*N* (%)	*N* (%)	
Sex			
Male	20 (80.0)	12 (48.0)	0.04
Female	5 (20.0)	13 (52.0)	
Ethnicity			
Chinese	15 (60.0)	12 (48.0)	0.6
Malay	6 (24.0)	9 (36.0)	
Indian	4 (16.0)	4 (16.0)	
Vaginal delivery	18 (72.0)	16 (64.0)	0.8
Cesarean delivery	7 (28.0)	9 (36.0)	
Maternal education level			
Less than 12 years	8 (33.3)	16 (64.0)	0.046
At least 12 years	16 (66.7)	9 (36.0)	
Family history of allergy	15 (65.2)	13 (65.0)	0.1
No family history of allergy	8 (34.8)	7 (35.0)	
Staying in same house since birth of child	11 (44.0)	10 (40.0)	0.9
Moved house since birth of child	14 (56.0)	15 (60.0)	
No pets at home	21 (84.0)	21 (84.0)	0.9
Having pets at home	4 (16.0)	4 (16.0)	
Number of days since last cleaning of home^a^	1.8 ± 0.4	1.6 ± 0.3	0.6
Number of days since last cleaning of sofa^a^	12.6 ± 3.2	33.8 ± 13.0	0.1
Number of days since last change of mattress cover^a^	9.3 ± 1.8	12.6 ± 18.5	0.4

^a^mean ± standard error mean

There were no significant differences in ethnicity, mode of delivery, family history of allergy and pet ownership between the groups (Table 1). The subjects from the allergic group cleaned the sofa and changed the bedsheet more regularly than those from the non-allergic group although it did not reach statistical significance (Table 1).

### Bacterial communities

**Fig. 1:**
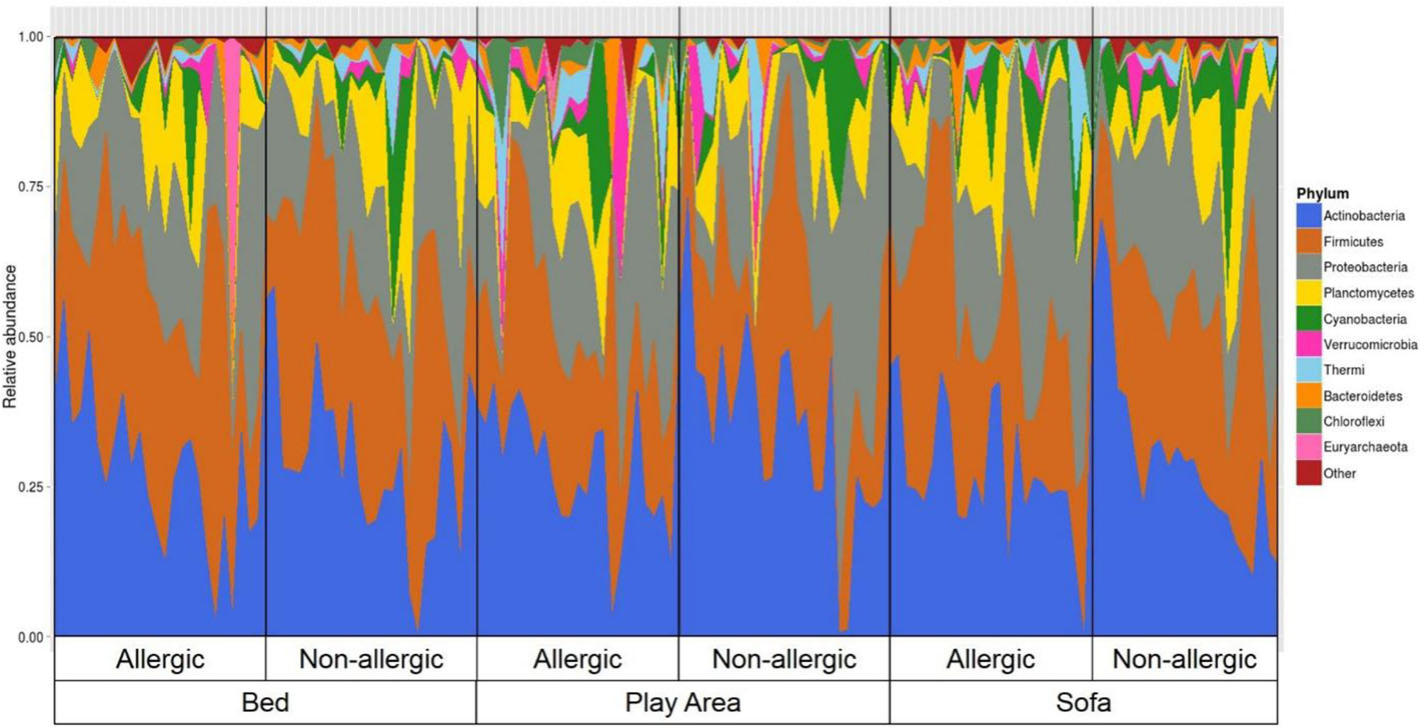
The overall phyla composition of individual dust samples from the bed, play area, and sofa of allergic and non-allergic subjects. The individual dust samples were grouped based on their location, followed by allergic status on the X-axis, and relative abundance on the Y-axis. The bacteria phyla with average relative abundance of > 7% across all samples or > 10% in at least 1 sample were represented. The dust samples contained primarily of *Actinobacteria*, *Firmicutes*, *Proteobacteria*, and *Planctomycetes*

**Fig. 2:**
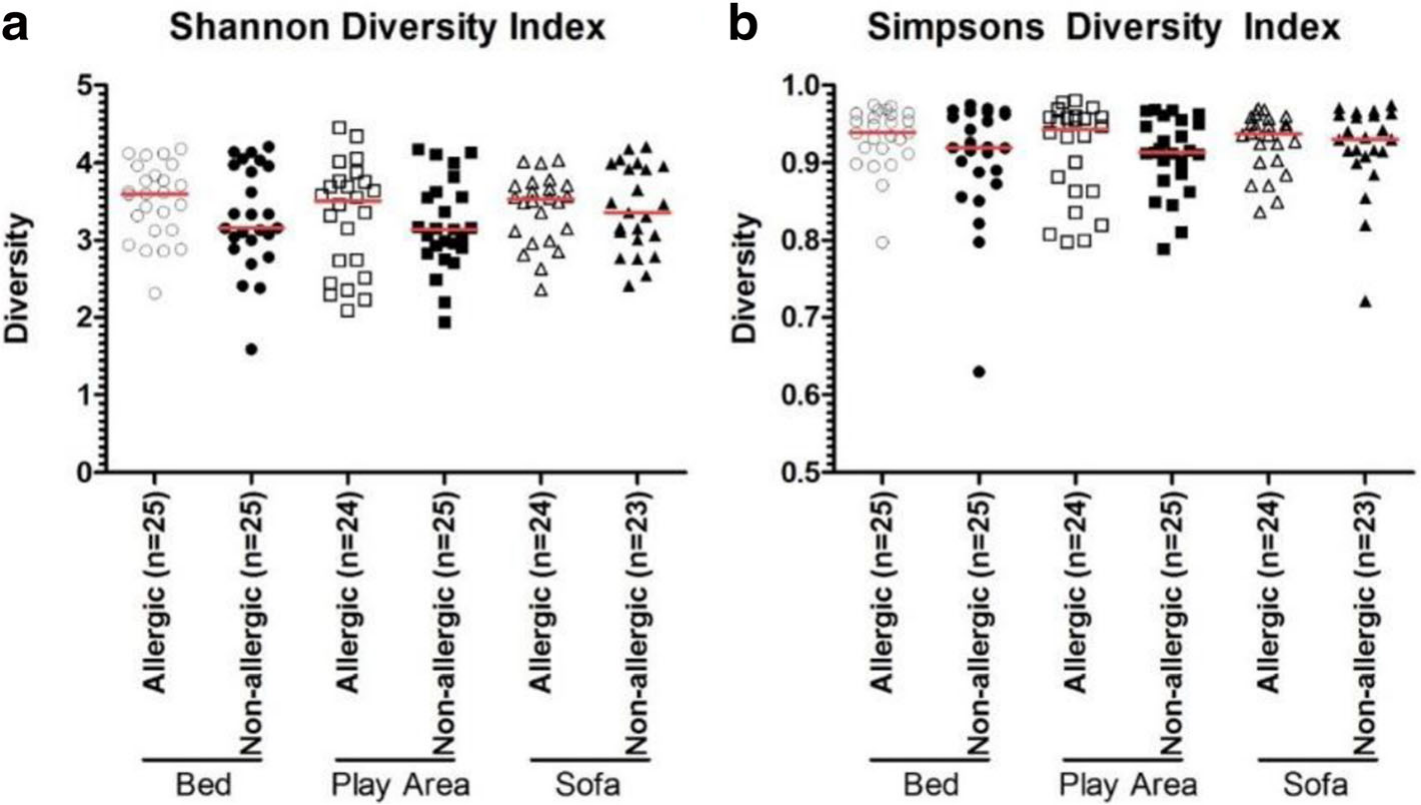
Bacterial diversity among dust samples from the bed (○), play area (□), and sofa (∆) of allergic (open) and non-allergic (close) subjects. Bacterial diversities were represented by Shannon Index (**a**) and Simpsons Index (**b**). Red line represents the median of each group. There were no difference in bacterial diversity between bed dust samples of allergic subjects and non-allergic subjects using Mann-Whitney U test. Similar trend was observed for play area and sofa samples

16S rRNA gene sequencing was performed on 146 dust samples only as 3 subjects did not have a sofa during the time of collection and 1 play area dust sample did not yield any visible PCR product on agarose gel electrophoresis. Sequencing of remaining 146 dust samples yielded on average of 212,741 V3-V4 raw sequence reads, with 88.2% of the sequence reads classified to the genus level. To assess if the sequencing depth was sufficient to cover the diversity of the samples, rarefaction curves were plotted showing the number of reads in each sample against the number of observed OTUs or Shannon diversity indices. Analysis of the rarefaction curves showed that the sequencing depth was sufficient (Additional file 1: Figure S1). The sequence reads were classified into 29 phyla, 63 classes, 123 orders, 276 families, and 860 genera, and only 11.8% of the sequences were not classified at the genus level. The most abundant phyla were Actinobacteria (29.8%), Firmicutes (27.7%), and Proteobacteria (22.4%, Fig. 1). The most abundant families were *Corynebacteriaceae* (9.4%), *Veillonellaceae* (9.3%), and *Staphylococcaceae* (7.6%). There was no significant difference in Shannon or Simpson’s diversity indices of bed dust samples of the allergic subjects and non-allergic subjects. Similar results were observed for the play area and sofa dust samples (Fig. 2).

To examine the role of bacterial composition of house dust samples in allergic sensitization, principal coordinate analysis (PCoA) based on Bray-Curtis dissimilarities was performed. PCoA displayed no distinct clustering effects of house dust samples of allergic subjects and non-allergic subjects (Additional file 1: Figure S2a). Similar results were observed for bed dust samples, play area dust samples, or sofa dust samples of allergic subjects and non-allergic subjects (Additional file 1: Figure S2b). These results indicate that the relative abundance of major bacterial taxonomic groups was similar between the house dust samples of allergic and non-allergic subjects.

**Fig. 3:**
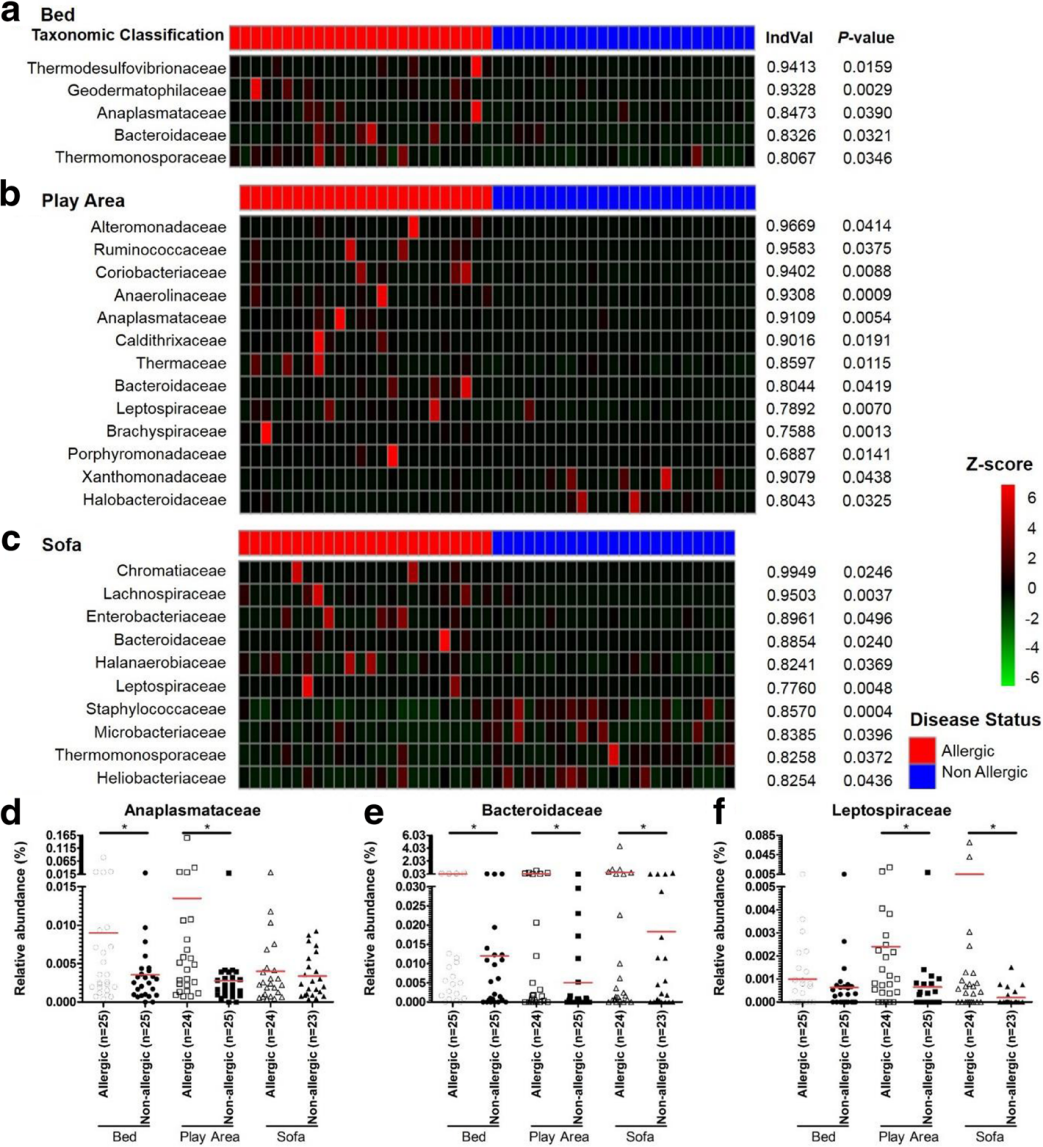
Relative abundance of bacterial families that were significantly different in the house dust samples of allergic subjects as compared to non-allergic subjects. Relative abundance of bacterial families in house dust samples from the bed (**a**), play area (**b**), and sofa (**c**) of allergic and non-allergic subjects were transformed into Z-score and represented using heat maps. Raw relative abundance of *Anaplasmataceae* (**d**), *Bacteroidaceae* (**e**), and *Leptospiraceae* (**f**) in the house dust samples from the bed (○), play area (□), and sofa (∆) of allergic (open) and non-allergic (close) subjects were represented using dot plot. Red line represents the mean of each group. Red line represents the mean of each group. Asterisk (*) represents significant difference of IndVal value > 0.6 and *P*-value < 0.05

To identify specific bacterial families in house dust samples that correlate with the allergic status of the subject, differences in relative abundance of bacterial families in house dust samples of allergic and non-allergic subjects were compared using relative indicator value analysis (IndVal). Bacterial families that were significantly different in the house dust samples of allergic and non-allergic subjects were identified, and their relative abundance were represented using heat-map (Fig. 3a, b, and c). Interestingly, relative abundance of *Anaplasmataceae*, *Bacteroidaceae*, and *Leptospiraceae* were significantly higher in dust samples of allergic subjects as compared to non-allergic subjects in 2 or more dust sampling locations.

Relative abundance of *Anaplasmataceae* was significantly higher in the bed dust samples of allergic subjects (Mean ± SEM, 0.00900 ± 0.00326) as compared to the non-allergic subjects (0.00354 ± 0.000838; Fig. 3d). Relative abundance of *Anaplasmataceae* was also significantly higher in the play area dust samples of allergic subjects (0.0135 ± 0.00645) as compared to the non-allergic subjects (0.00277 ± 0.000756). There was a trend of higher relative abundance of *Anaplasmataceae* in the sofa dust samples of allergic subjects (0.00402 ± 0.00101) as compared to the non-allergic subjects (0.00338 ± 0.000596), albeit without statistical significance.

In all 3 locations, the relative abundance of *Bacteroidaceae* was significantly higher in the house dust samples of allergic subjects (bed 0.0444 ± 0.0157; play area 0.0529 ± 0.0255; sofa 0.280 ± 0.183) as compared to the non-allergic subjects (bed 0.0120 ± 0.00418; play area 0.00503 ± 0.00194, sofa: 0.0183 ± 0.00858; Fig. 3e). Relative abundance of *Leptospiraceae* was significantly higher in the play area and sofa dust samples of allergic subjects (play area 0.002403 ± 0.000877; sofa 0.00518 ± 0.00328), as compared to the non-allergic subjects (play area 0.000652 ± 0.000348; sofa 0.000194 ± 0.00008; Fig. 3f). There was a trend of higher relative abundance of *Leptospiraceae* in the bed dust samples of allergic subjects (0.000993 ± 0.000261) as compared to the non-allergic subjects (0.000636 ± 0.000218), albeit without statistical significance.

### Indoor allergens

To investigate the role of indoor allergens in allergic sensitization, allergens were extracted from house dust samples of allergic and non-allergic subjects and measured using ELISA or MARIA assays. A total of 147 house dust samples were measured for *Der f* 1, *Der p* 1, mite group 2, tropomyosin, *Can f* 1, and *Fel d* 1 as 3 subjects did not have a sofa during the time of collection. Only 138 and 116 house samples were measured for *Blo t* 5 and tropomyosin respectively, as remaining samples collected were insufficient for the assays. The prevalence of mite group 2 (99.3%), tropomyosin (99.1%), *Der p* 1 (98.6%), and *Blo t* 5 (96.4%) were higher than *Fel d* 1 (59.2%), *Der f* 1 (53.1%), and *Can f* 1 (36.7%). The dust samples contained on average 66.9 ng/g of *Der f* 1, 218 ng/g of *Der p* 1, 178 ng/g of *Blo t* 5, 2676 ng/g of mite group 2, 287 ng/g of tropomyosin, 18.5 ng/g of *Can f* 1, and 6.4 ng/g of *Fel d* 1.

**Fig. 4:**
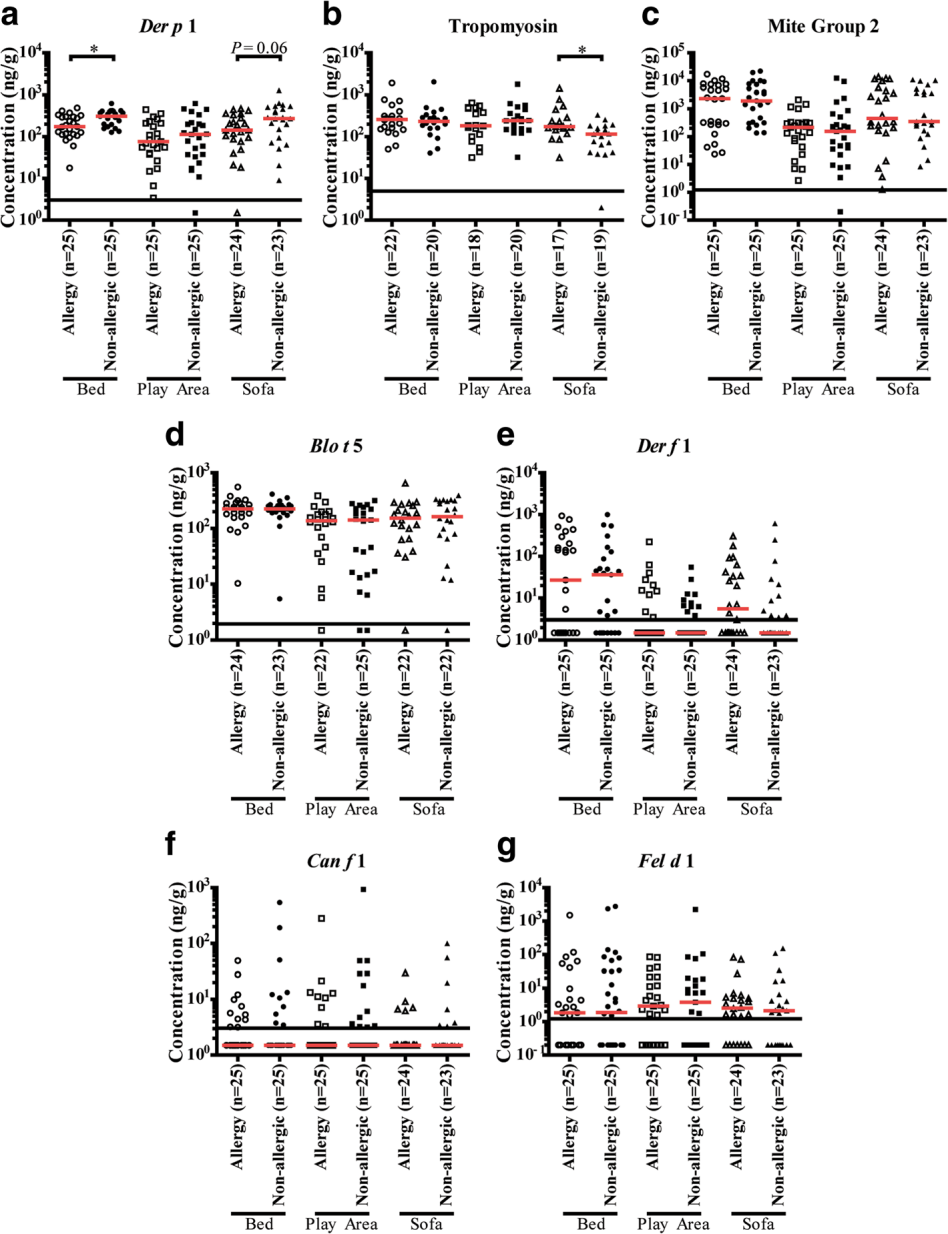
Concentration of indoor allergens, (**a**) Der p 1 (**b**) Tropomyosin (**c**) Mite Group 2 (**d**) Blo t 5 (**e**) Der f 1 (**f**) Can f 1(**g**) Fel d 1 of dust samples from the bed (○), play Area (□), and sofa (∆) of allergic (open) and non-allergic (close) subjects. Red line represents the median of each group. Dotted lines represent the lower limit of detection of each allergen. Asterisk (*) represents significant difference of *P*-value < 0.05

The concentration of *Der p* 1 was significantly lower in bed dust samples of allergic subjects (Median [Interquartile range], 174 ng/g [115–299 ng/g]) as compared to non-allergic subjects (309 ng/g [201–400 ng/g]; *P* < 0.05; Fig. 4a). The concentration of *Der p* 1 was also lower in sofa dust samples of allergic subjects (143 ng/g [84.1–292 ng/g]) as compared to non-allergic subjects (271 ng/g [122–482 ng/g]), albeit with borderline significance (*P* = 0.0626). There was no difference in *Der p* 1 concentration in play area dust samples between allergic subjects and non-allergic subjects.

Interestingly, the concentration of tropomyosin was significantly higher in sofa dust samples of allergic subjects (175 ng/g [145–284 ng/g]) as compared to non-allergic subjects (116 ng/g [52.8–170 ng/g]; *P* < 0.05; Fig. 4b). Twenty (83.3%) allergic subjects eat on the sofa compared to 16 (69.6%) of non-allergic subjects (*p* = 0.3, data not shown) There was no difference in the concentration of tropomyosin in bed or play area dust samples between allergic and non-allergic subjects. There was no difference in the concentration of other allergens in house dust samples between allergic subjects and non-allergic subjects. We did not observe any correlation between microbiota and allergen levels (data not shown).

Since tropomyosin can be derived from crustacean sources, such as shrimp, crab, and lobster, or non-crustacean sources, such as dust mites (*Blo t*, *Der f,* and *Der p*), the relationship between concentration of tropomyosin, and concentration of *Blo t* 5, *Der f* 1, or *Der p 1* in sofa dust samples were examined using Spearman rank-order correlation analysis. The analysis shows that there was no significant correlation between concentration of tropomyosin and concentration of *Blo t* 5, *Der f* 1, or *Der p* 1 in sofa dust samples of allergic subjects (Additional file 1: Figure S3a, b, and c). Similar results were observed for sofa dust samples of non-allergic subjects (Additional file 1: Figure S3d, e, and f). Therefore higher concentration of tropomyosin observed in sofa dust samples of allergic subjects as compared to non-allergic subjects was unlikely to be due to house dust mites.

## Discussion

In this study we found that the most abundant phyla in house dust were Actinobacteria*,* Firmicutes and Proteobacteria which has also been reported by a European study that included 102 children from 6 to 12 years of age staying in rural areas [31]. In addition, the presence of these bacteria has also been reported on skin microbiota, suggesting a link between environmental and skin microbiota [32]. We also observed increased abundance of the bacteria families, *Bacteroidaceae, Anaplasmataceae* and *Leptospiraceae* across all 3 sites from houses of allergic subjects although there is no difference in bacteria diversity or major bacterial taxonomic groups (relative abundance of > 1%) between houses of allergic and non-allergic subjects. In agreement with our observations, a study by Ciacco et al. also reported that genus richness did not differ in the houses of asthmatics and non-asthmatics. Instead, different bacteria were in found in higher abundance in the houses of asthmatic patients [19].

Interestingly, all three of these bacterial families are gram-negative. Another study analyzing dust samples from Russian and Finnish Karelian also reported a higher proportion of Gram-negative bacteria in Finnish households, where the risk of atopy in childhood was nearly fourfold higher [25]. Similarly, another birth cohort study in New York City measured the endotoxin levels in dust samples taken from the houses of 12 or 36 month old children of African-American or Dominican descent and they found a positive association between higher endotoxin levels with wheeze at 2 years [33]. Endotoxins are polysaccharides found on the outer membrane of Gram-negative bacteria, and are usually used to assess gram-negative bacterial load. Another study in Boston also reported that endotoxin exposure could independently cause elevated risk of wheeze and recurrent wheeze in 1 year old children, with a family history of asthma or allergy [34].

On the contrary, a study in Germany found that higher level of endotoxin in the mattress dust was associated with a significant reduction in the risk of atopic diseases such as hay fever, atopic sensitization, atopic asthma, and atopic wheeze in children. [35]. Likewise, another study done in Germany also reported an inverse relationship between endotoxin levels and atopic dermatitis in children aged 2–4 years [36]. However, not all gram-negative bacteria are lipopolysaccharide- (LPS) positive. A study by Borjesson et al. showed that some members of the *Anaplasmataceae* family are LPS-negative and these LPS-negative gram-negative bacteria were shown to elicit different proinflammatory responses as compared to the LPS-positive gram-negative bacteria while another study also showed that *N.helminthoeca*, another member of the *Anaplasmataceae* family, has no ability to synthesise LPS. [37, 38] . Furthermore, a study by Patra et al also demonstrated that the structure of LPS differs between pathogenic and intermediately pathogenic species of *Leptospiraceae* [39]. These studies suggest that the production and structure of endotoxin differ between different species of gram-negative bacteria.

Interestingly, a study in a Japanese population found that *Bacteroidaceae* was found at higher levels in gut microbiota of allergic subjects at ages 1 and 2 months, as compared to the non-allergic subjects [40]. This suggests a possible link between environmental microbiota and gut microbiota. Another study also found that the presence of *Bacteroides* was associated with the extent of atopic sensitization before weaning [41]. Infants with atopic eczema had significantly higher proportion of *Bacteroides* prior to weaning. Typically, *Bacteroides* tend to increase in population in the infant’s gut after weaning. However, Lynch et al found that members of the Bacteroidetes phyla, were significantly increased in the first-year dust samples of the non-allergic group, suggesting possible protective effects [14].

As shellfish allergy is most prevalent at this age group of 5 years in Singapore [42, 43], we measured tropomyosin, the major allergen in shellfish, in this study and, we found that the concentration of tropomyosin was elevated in the sofa dust samples of allergic subjects as compared to non-allergic subjects. In addition, within the allergic group, concentration of tropomyosin is also higher in houses of subjects sensitized to shellfish allergens compared to the nonsensitized subjects. One possible reason for this increased tropomyosin concentration could be due to the presence of crustacean (shrimp, crab, lobster) allergens in homes, which could lead to sensitization via cutaneous contact. A study by Fox et al also shows that environmental exposure to higher levels of peanut allergen increases the risk of developing peanut allergy [44]. Dust samples with high levels of peanut allergens have also been found to be biologically active and can activate basophils in a dose dependent manner in children with peanut allergy [45]. Besides shellfish allergens, another possible source of tropomyosin may also be from cockroach allergens. We noticed lower concentrations of *Der p* 1 on the bed and sofa dust samples collected from the houses of allergic subjects compared to the non-allergic subjects and this can possibly be due to the higher frequency of cleaning of sofa as well as changing of bedsheets by the allergic subjects, although this did not reach statistical significance. Similarly, the comparable levels of *Der p* 1 from the dust collected in the play areas could be due to a similar cleaning frequency of the houses by the allergic and non-allergic subjects.

One of the strengths of this study is that the GUSTO study is the first in Asia to study the relation of both microbial and allergens exposure on allergic outcomes. To the best of our knowledge, there is only one other study in the United States that studied the effect of a combination of bacterial/allergen exposure to atopy [14]. In addition, we used an objective assessment of environmental allergen levels by assaying it from dust instead of a questionnaire-based approach. Besides that, instead of measuring endotoxin levels as an indirect assessment of bacterial levels, we employed 16S rRNA sequencing techniques to identify specific bacteria communities. Seasonal variation is likely to affect microbiota and allergen concentration, however in Singapore, a tropical climate with constant UVB radiation is present year-round due to our geographical location on the equator. However, as this is a cross-sectional study, we are unable to determine causality and if house dust composition is affected by allergic status. Besides that, we did not collect information on number of occupants in the house which may potentially also affect microbiota and allergen levels. In addition, mode of delivery is known to affect allergic outcomes, however we did not observe differences in mode of delivery between the allergic and non-allergic subjects in our study, this is likely due to our small sample size. Another limitation is the low amounts of dust samples collected which did not allow us to analyse the levels of more food allergens such as peanut allergens.

In conclusion, we found a differential microbiota and allergen profile between houses of allergic and non-allergic subjects.

## Additional file

Additional file 1: **Figure S1.** Rarefaction curves showing the number of reads per sample against observed operational taxonomic unit (OTU) or Shannon diversity indices of each dust sample. **Figure S2.** Principal coordinates plots of house dust samples of allergic and non-allergic subjects based on Bray-Curtis dissimilarities. **Figure S3.** Correlation analysis of the concentrations of tropomyosin and house dust mites of dust samples from Sofa of allergic subjects and non-allergic subjects. (DOCX 1027 kb)

## Abbreviations

GUSTO: Growing Up in Singapore Towards Healthy Outcomes
ISAAC: International Study of Asthma and Allergies in Childhood
LPS: Lipopolysaccharide
SPT: Skin prick testing
