# Supplementary material for: Comparison of microbiota and allergen profile in house dust from homes of allergic and non-allergic subjects- results from the GUSTO study
Source: World Allergy Organ J. 2018 Dec 5;11(1):37. doi: 10.1186/s40413-018-0212-5 (PMC6280478; doi:10.1186/s40413-018-0212-5)
Supplement: Supplementary file 1 — Figure S1. Rarefaction curves showing the number of reads per sample against observed operational taxonomic unit (OTU) or Shannon diversity indices of each dust sample. Figure S2. Principal coordinates plots of house dust samples of allergic and non-allergic subjects based on Bray-Curtis dissimilarities. Figure S3. Correlation analysis of the concentrations of tropomyosin and house dust mites of dust samples from Sofa of allergic subjects and non-allergic subjects. (DOCX 1027 kb) [file 40413_2018_212_MOESM1_ESM.docx]

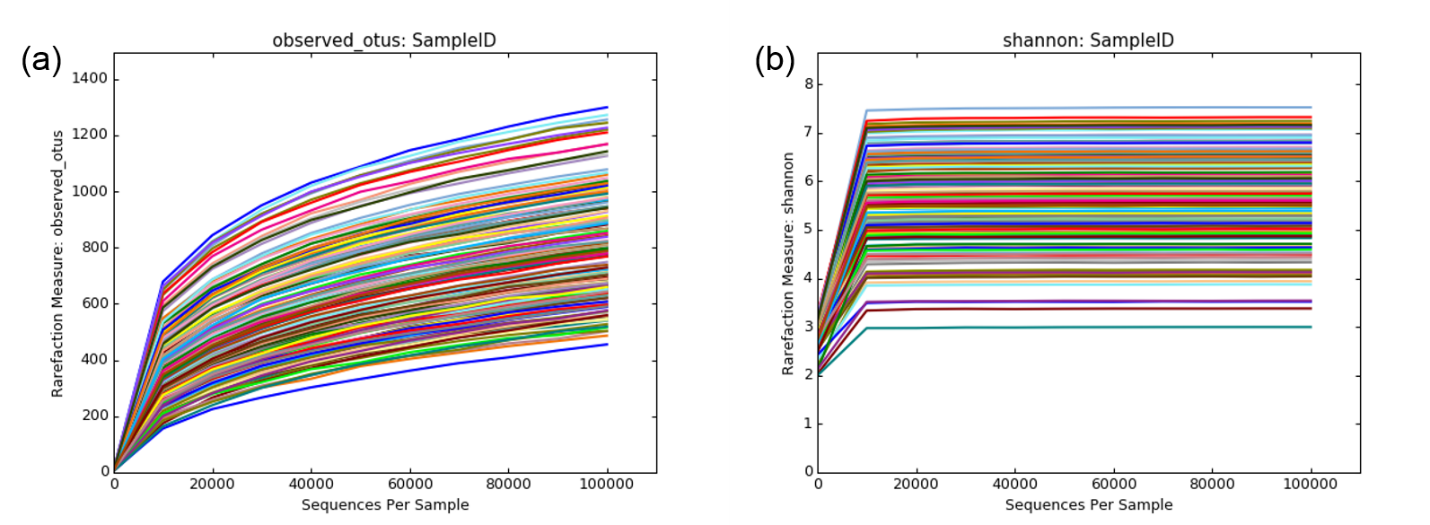


**Figure S1: Rarefaction curves showing the number of reads per sample against observed operational taxonomic unit (OTU) or Shannon diversity indices of each dust sample.**


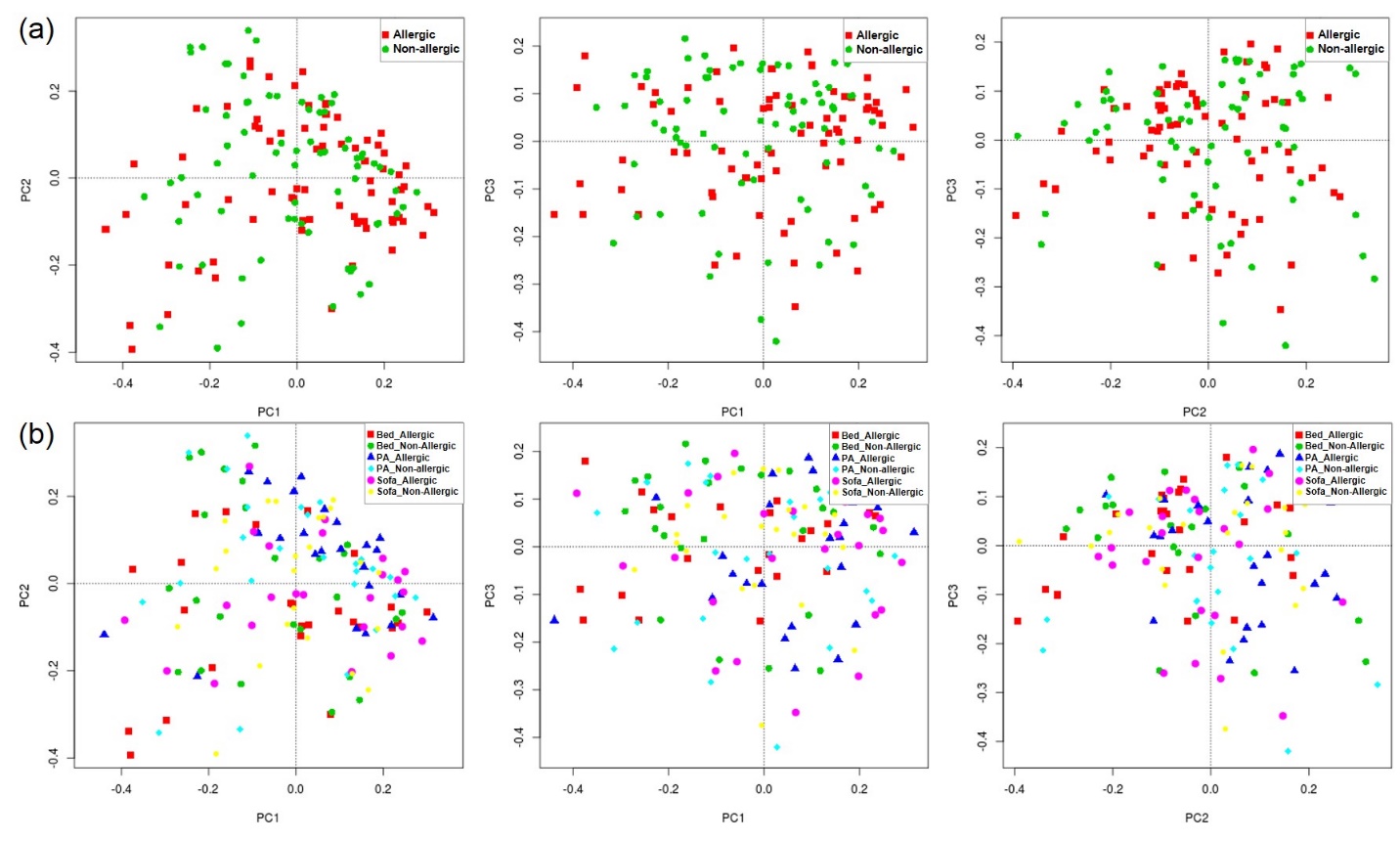


**Figure S2: Principal coordinates plots of house dust samples of allergic and non-allergic subjects based on Bray-Curtis dissimilarities.** The axes shown were rotated to PC1-10.03%, PC2-8.05%, PC3-5.69%. There was no distinct clusters of house dust samples of allergic and non-allergic subjects (a). There was also no distinct clusters of bed, play area (PA), and sofa dust samples of allergic and non-allergic subjects (b).


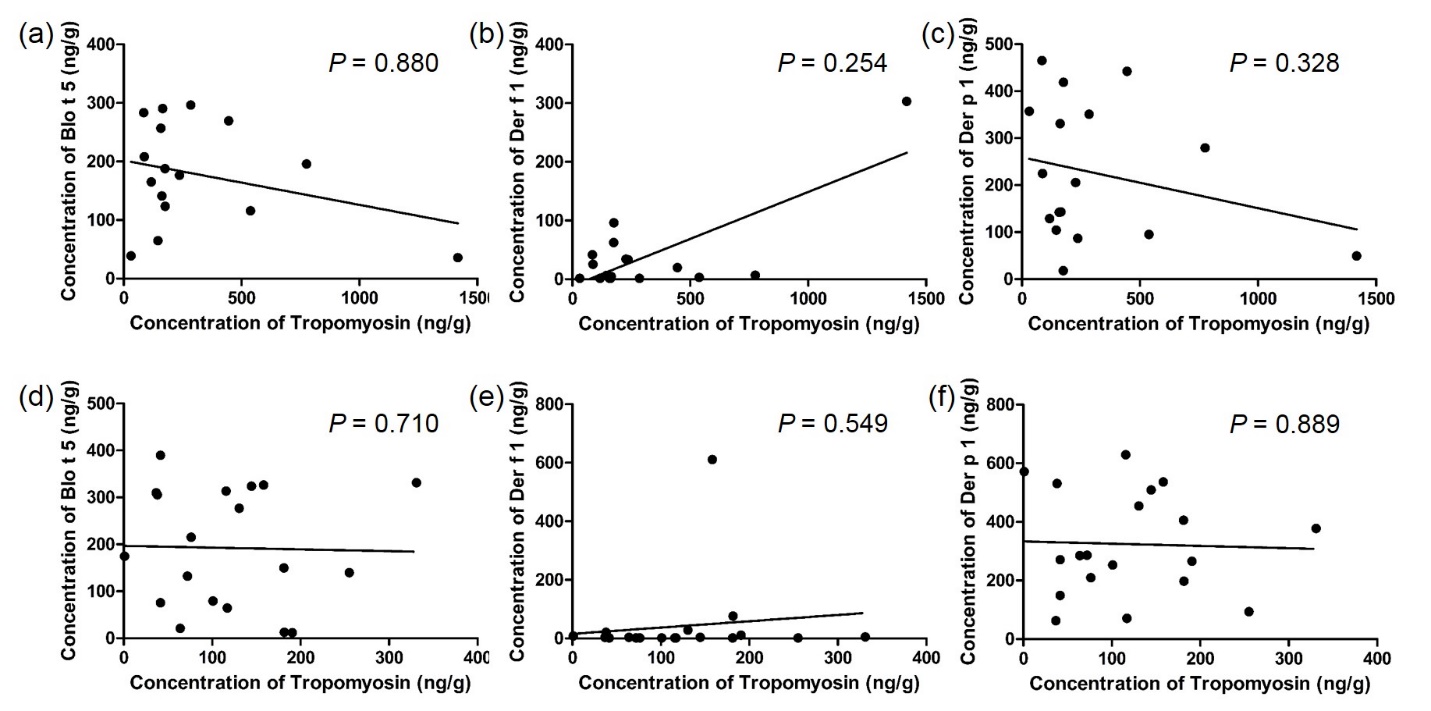


**Figure S3: Correlation analysis of the concentrations of tropomyosin and house dust mites of dust samples from Sofa of allergic subjects and non-allergic subjects.** There was no significant correlation between concentration of tropomyosin and Blo t 5, Der f 1, or Der p 1 in the sofa dust samples of allergic subjects (a-c) and non-allergic subjects (d-f) using Spearman’s rank-order correlation analysis.
